# Artificial Digestion of Polydisperse Copper Oxide Nanoparticles: Investigation of Effects on the Human In Vitro Intestinal Co-Culture Model Caco-2/HT29-MTX

**DOI:** 10.3390/toxics10030130

**Published:** 2022-03-07

**Authors:** Jevin Büttner, Thomas Schneider, Martin Westermann, Michael Glei

**Affiliations:** 1 Department of Applied Nutritional Toxicology, Institute of Nutrition, Friedrich Schiller University Jena, Dornburger Str. 24, 07743 Jena, Germany; thomas.schneider@uni-jena.de (T.S.); michael.glei@uni-jena.de (M.G.); 2 Department of Cardiothoracic Surgery, Jena University Hospital, Am Klinikum 1, 07747 Jena, Germany; 3 Electron Microscopy Center, Jena University Hospital, Ziegelmühlenweg 1, 07743 Jena, Germany; martin.westermann@uni-jena.de

**Keywords:** static in vitro digestion, copper oxide nanoparticles, intestinal barrier integrity, cell viability, apoptosis, delivered dose, mono-/co-culture

## Abstract

Copper oxide nanoparticles (CuO-NP) are increasingly used in consumer-related products, which may result in increased oral ingestion. Digestion of particles can change their physicochemical properties and toxicity. Therefore, our aim was to simulate the gastrointestinal tract using a static in vitro digestion model. Toxic properties of digested and undigested CuO-NP were compared using an epithelial mono-culture (Caco-2) and a mucus-secreting co-culture model (Caco-2/HT29-MTX). Effects on intestinal barrier integrity, permeability, cell viability and apoptosis were analyzed. CuO-NP concentrations of 1, 10 and 100 µg mL^−1^ were used. Particle characterization by dynamic light scattering and transmission electron microscopy showed similar mean particle sizes before and after digestion, resulting in comparable delivered particle doses in vitro. Only slight effects on barrier integrity and cell viability were detected for 100 µg mL^−1^ CuO-NP, while the ion control CuCl_2_ always caused significantly higher adverse effects. The utilized cell models were not significantly different. In summary, undigested and digested CuO-NP show comparable effects on the mono-/co-cultures, which are weaker than those of copper ions. Only in the highest concentration, CuO-NP showed weak effects on barrier integrity and cell viability. Nevertheless, a slightly increased apoptosis rate indicates existing cellular stress, which gives reason for further investigations.

## 1. Introduction

Copper oxide nanoparticles (CuO-NP) are characterized by their versatility in numerous applications, which is why they are used primarily in technical sectors, such as the semiconductor industry, the shipping industry or petroleum production due to their usefulness as pigments or catalysts [1,2,3]. Furthermore, they are increasingly found in consumer-related products, such as textiles [4], food packaging [5], intrauterine devices [6] or wood preservatives [7], mainly due to their antimicrobial, antifungal and biocidal properties.

Their frequent use greatly increases the likelihood of human exposure to CuO-NP, which can be explained primarily by the inevitable release of nanoscale material into the environment [8,9]. For example, their use can lead to undesirable contamination of drinking water and natural water bodies [10]. In turn, contamination of water bodies may result in increased accumulation of nanoparticles in aquatic life, such as fish or shellfish, which would greatly increase the risk of human exposure to CuO-NP through their consumption [11]. In addition to ingestion through contaminated foods, indirect exposure may also occur through the detachment, seepage or leaching of CuO-NP from packaging [8,12]. Therefore, after oral ingestion of these foods, an increased exposure of the human gastrointestinal tract also occurs [3]. Due to the increased risk of ingesting CuO-NP via food contamination, a risk assessment is essential to accurately determine potential adverse effects on consumer health.

In principle, the toxicity of nanomaterials is influenced by their respective physicochemical properties [13]. Among the frequently discussed parameters responsible for the various biological effects and behaviors of CuO-NP in solution are particle size, morphology, surface charge, solubility and interactions of the NP with other macromolecules in the respective medium [14,15,16]. Due to their sometimes highly increased reactivity, CuO-NP can have negative effects on cells and organs. Thus, CuO-NP exhibited various toxic and barrier integrity-lowering effects in in vitro intestinal and epithelial cell models [16,17,18,19,20,21,22,23,24,25,26,27,28]. Moreover, toxic effects of CuO-NP on the liver, spleen and kidneys have already been described in mouse models [29,30,31]. In this context, the damaging effects of CuO-NP are due to different mechanisms, which mainly include the triggering of oxidative stress [16,21,32,33,34,35], damage to the genetic material [33,36,37,38] and the induction of inflammatory processes [35,39,40,41].

In vitro studies on the toxicity of CuO-NP have been predominantly performed on the well-characterized intestinal cell model Caco-2, with toxic effects occurring both in undifferentiated cells of colorectal origin [2,42] and in the differentiated variant exhibiting characteristics of human enterocytes [2,43]. Moreover, the negative influence of CuO-NP has also been demonstrated on much more complex cell culture models, such as a 3D gastrointestinal model with microfold and mucus secreting cells [3] or a model for mapping the intestinal–liver axis [12], which can already better represent the actual in vivo situation. In summary, there is some evidence that oral uptake of CuO-NP could be linked to various adverse health effects in vivo. However, all the in vitro studies conducted so far to assess the risk of CuO-NP have ignored one important fact: the influence of the digestive process itself on the physicochemical properties of the ingested NP. However, it is precisely this digestion that can have a significant influence on the toxicity of the nanomaterial, which is why the European Food Safety Authority emphasizes their consideration in its guidance on nanoparticle risk assessment [13].

During the digestive process, orally ingested CuO-NP are exposed to various factors that can potentially modify their physicochemical properties and thus influence their harmful potential. It has already been shown in several studies that particle size, aggregation/agglomeration behavior, ion release, generation of reactive oxygen species (ROS) and cellular uptake of different NPs can be affected during the digestion process and that, consequently, the toxicity of these NP can also be modified due to the digestion process [14,44,45,46,47,48,49]. A parameter of particular significance is the strong pH difference in the digestive tract, whose great influence on metal oxide nanoparticles has already been shown [50]. However, other parameters such as temperature, transit time and organic/inorganic components as well as the food matrix itself may also play a major role here in processes such as dissociation, agglomeration and structural and chemical changes. Only one recent paper has at least included the influence of digestive enzymes in their CuO-NP toxicity studies, albeit without considering the other parameters in the digestive juices as mentioned before [51].

The aim of the present study was therefore to investigate the influence of the digestion process on polydisperse CuO-NP by using a static in vitro digestion approach. After a detailed particle characterization, the toxicity of CuO-NP digested in this way should be compared with the effects of pristine (undigested) CuO-NP. As relevant toxicological endpoints, cellular uptake, effects on barrier integrity, influences on cell viability and apoptosis induction of CuO-NP were investigated. To consider potential attenuating effects of intestinal mucosa on toxicity, a co-culture model with Caco-2 and mucus-secreting HT29-MTX cells was used in addition to the classical Caco-2 monoculture. Furthermore, a particle transport model (distorted grid) for particles in fluids was applied, allowing simulation of nanoparticle behavior in in vitro experiments and calculation of the delivered particle doses.

## 2. Materials and Methods

### 2.1. Nanoparticles

We purchased the investigated metal oxide nanopowder (No. 544868 copper (II) oxide) from Sigma–Aldrich (Schnelldorf, Germany) and its associated ion control (CuCl_2_) from VWR International LLC (Radnor, PA, USA).

### 2.2. Reagents

Non-essential amino acids (NEA), Dulbecco’s Modified Eagle’s Medium (DMEM) and DMEM without phenol red were purchased from Biochrom GmbH, Berlin, Germany. Bovine serum albumin (BSA), dimethyl sulfoxide (DMSO), ethanol, hydrochloric acid, urea, 4-(2-hydroxyethyl)-1-piperazineethanesulfonic acid (HEPES), potassium chloride, potassium dihydrogen phosphate, disodium hydrogen phosphate, sodium chloride and sodium hydrogen carbonate were obtained from Carl Roth GmbH & Co. KG, Karlsruhe, Germany. Alcian blue, Triton X-100, 4′6-diamidino-2-phenylindole (DAPI), fluorescein isothiocyanate dextran (FITC-dextran), porcine bile, magnesium chloride, porcine mucin, pancreatin, penicillin/streptomycin, pepsin, trypsin, trypsin/ethylenediaminetetraacetic acid (EDTA), calcium chloride and α-amylase came from Sigma–Aldrich GmbH, Germany. Chloroform and acetic acid were from Thermo Fisher Scientific, Waltham, MA, USA. Foetal bovine serum (FBS) was purchased from PAN-Biotech GmbH, Aidenbach, Germany. Annexin V was obtained from Beckman Coulter Corp., Brea, CA, USA. Ethylene glycol-bis (β-aminoethyl ether)-N,N,N′,N′-tetraacetic acid (EGTA) came from AppliChem GmbH, Darmstadt, Germany. Whole milk powder was purchased from J.M. Gabler-Saliter Milchwerk GmbH & Co. KG, Obergünzburg, Germany.

### 2.3. Preparation of Nanoparticle Dispersions and Ion Control

**Figure 1:**
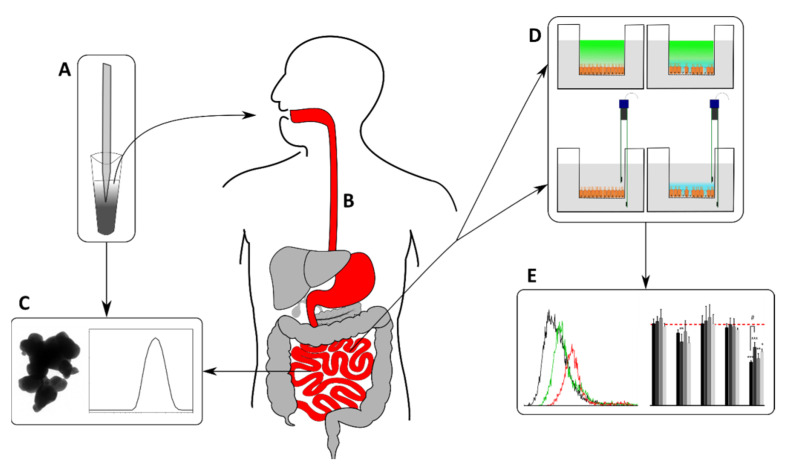
Schematic illustration of the applied experimental setup. (**A**) Particle dispersion preparation by ultrasonication, (**B**) artificial digestion using a static in vitro approach, (**C**) dispersion characterization by transmission electron microscopy and dynamic light scattering, (**D**) intestinal barrier integrity measurements of transepithelial electrical resistance and permeability of FITC-dextran, (**E**) analysis of cell viability and induction of apoptosis by flow cytometry. The statistical differences between the samples and the NC are marked as * *p* < 0.05, ** *p* < 0.01 and *** *p* < 0.001, and the statistical difference between the digested/undigested samples is marked as # *p* < 0.05.

We generated stable particle dispersions (Figure 1A) according to the modified standard operating procedure from the NanoGenoTOX protocol [52] and as described by DeLoid et al. [53]. For undigested CuO dispersions, we prepared stock solutions with a concentration of 6.67 mg CuO nanopowder in 1 mL dispersion media (0.05% *w/v* sterile-filtered bovine serum albumin in water containing 0.5% *v/v* ethanol for pre-wetting [52]). In-probe sonication was used for disruption of larger particle agglomerates/aggregates. For this, we filled a glass rosette cell (Bandelin, RZ2) with 40 mL of our sample. We plunged an ultrasonic homogenizer (Bandelin Sonopuls HD 2070) 1 cm into the sample solution, which was cooled down during sonication by a surrounding ice-cold water bath. The critical delivered sonication energy (DSEcr), where no further decrease in the mean particle size can be detected, was investigated as published in our previous work [54]. We applied 720 J mL^−1^ to our CuO stock dispersions by direct ultrasonication. Working solutions of each sample in cell culture media (supplemented with 10% FBS) were diluted to reach final CuO concentrations of 1 to 100 µg mL^−1^ (1.3 × 0^−5^ to 1.3 × 10^−3^ M), followed by vortexing at maximum speed (3000 rpm) for 30 s. We chose the aforementioned CuO-NP concentrations based on the benchmark dose (BMD20) mentioned by Ude et al. [2], which roughly corresponds to 1.3 × 10^−4^ M and based on the concentrations of Titma et al. [26] as well as Henson et al. [51]. We chose equal concentrations (1.3 × 10^−5^ M to 1.3 × 10^−3^ M) of CuCl_2_ as the ion control for our experiments. Thereby the same amount of Cu ions/atoms would be present in the corresponding solutions/dispersions. We analyzed the resulting particle dispersions by dynamic light scattering and transmission electron microscopy (TEM) for mean particle size directly after preparation. To test for size stability over time, we analyzed samples for 90 min in short intervals (7 min each) directly after preparation (stability data can be found elsewhere [54]). In addition to assess the particle stability over the course of the incubation, we tested the stability of CuO-NP dispersions after 24 h by dynamic light scattering as described below.

### 2.4. Static In Vitro Digestion Simulation

The artificial in vitro digestion of CuO-NP (Figure 1B) was carried out according to DIN 19738 2017 [55] and Sieg et al. [48] to approximate the physiological conditions that might influence orally administered CuO-NP. While stock solutions of the corresponding salt concentrations for each digestive phase were initially prepared, the enzymes were only added on the day of the in vitro digestion. Following the addition of salt solutions and enzymes, the pH values were adjusted with HCl and NaHCO_3_. After this step, the digestive juices were placed in a 37 °C water bath. Whole milk powder was used as a food matrix. The compositions of the digestive juices are described in DIN 19738 2017 [55] and can also be found in Table S1 of additional file 1.

Initially, 6.67 mg mL^−1^ CuO-NP were sonicated in artificial saliva by in-probe sonication as described above. For the highest sample concentration, 15 mL of this solution was then added to 1 g of whole milk powder and 41.6 µL α-amylase. The lower concentrations were diluted 10 times/100 times in artificial saliva and then added to a flask with the food matrix and α-amylase. Apart from the sonication, the same procedure was also applied for the digestion of CuCl_2_. The highest concentration of 1.3 × 10^−3^ M CuCl_2_, which is the equimolar concentration to 100 µg·mL^−1^ of CuO-NP, was then used either undiluted or diluted by 10 times/100 times in artificial saliva before adding it to the corresponding flask containing the milk powder and α-amylase. To simulate the mouth phase of digestion, the flasks were placed in a shaking water bath (120 rpm) and incubated for 5 min at 37 °C. The gastric phase was simulated by adding 35 mL of artificial gastric juice, adjusting the pH values to pH 2.0 and incubating the flasks for 2 h at 37 °C and 120 rpm. The pH values were periodically checked and adjusted to pH 2.0 as necessary. The last digestive step was simulated by adding 50 mL of artificial intestinal juice, adjusting the pH levels to 7.5 and incubating the CuO-NP for 3 h at 37 °C and 120 rpm. Again, the pH values were periodically checked and adjusted to 7.5 as necessary.

### 2.5. Particle Characterization

#### 2.5.1. Dynamic Light Scattering and Electrophoretic Mobility

We quantified the mean particle size and surface charge (Figure 1C) using a Malvern Zetasizer Nano ZS (Panalytical Instruments, Malvern, UK). To measure the mean particle size by dynamic light scattering (DLS), we analyzed the scattering intensity expressed as “mean size” (or dDLS) and the polydispersity index “PdI”. We determined the zeta potential as a parameter for particle surface charge by electrophoretic mobility. For both DLS and zeta potential, we analyzed three independent triplicates of each sample with three measurements and a duration of 10 s per run (the number of runs was set automatically) according to DIN-ISO-22412 2018 [56].

#### 2.5.2. Transmission Electron Microscopy

Image acquisition of nanoparticle samples was realized as previously described by Schneider et al. [54]. The particle dispersions were characterized by using transmission electron microscopy (TEM). For that process, we mounted freshly prepared particle dispersions onto Formvar-carbon filmed 400 mesh copper grids (Quantifoil, Großlöbichau, Germany), allowed them to air-dry and examined them in a Zeiss CEM 902 A electron microscope (Carl Zeiss AG, Oberkochen, Germany). We used a Sharp:Eye 2 k wide-angle slow-scan CCD camera and ImageSP v1.2.10.39 acquisition software (camera and software Tröndle TRS, Moorenweis, Germany). All images shown are representative of the analyzed samples.

#### 2.5.3. Determination of Delivered Doses by Transport Simulation

For undigested CuO particle dispersions, the delivered dose was calculated using the DG model following the standard operation procedure as described by DeLoid et al. [53]. The transport model required determination of the effective CuO density, which was determined in triplicate for three independently prepared CuO dispersions using the volumetric centrifugation method [54]. All relevant parameters for modeling the delivered dose of undigested CuO-NP are available in Table S2 of Supplementary Materials.

### 2.6. Cell Culture and Exposure

Caco-2 (passages 14–24) and HT29-MTX (passages 8–18) cells were cultured separately in 75 cm^2^ cell culture flasks (Greiner Bio-One GmbH, Frickenhausen, Germany) at 37 °C, 95% humidity and 5% CO_2_. The cells were maintained in DMEM supplemented with 10% FBS, 1% penicillin/streptomycin and 1% NEA. Prior to incubation, the cells were differentiated. For the mono-culture 50,000 Caco-2 cells were seeded into the apical compartment of a 12-well Transwell insert (Corning Inc., New York, NY, USA) with 500 µL cell culture medium. The basolateral compartments were filled with 1.5 mL of cell culture medium. For the co-culture, 40,000 Caco-2 cells and 10,000 HT29-MTX cells were seeded into the apical compartment in accordance with Stock et al. [57]. The cells were cultured at 37 °C, 95% humidity and 5% CO_2_ for 21 days. The cell culture medium in the apical and basolateral compartments was changed every other day. For incubation, the cell culture medium was removed from the apical compartment, and 500 µL of digested and undigested CuO-NP dispersions, diluted 1:10 in cell culture medium, were pipetted into the Transwell insert. After 24 h, the study endpoints were analyzed. The dilution factor of digested particles was determined in preliminary experiments to exclude cytotoxic concentrations of the digestive juice (DJ) itself (Supplementary Materials, Figures S1 and S2). For this, the influence of DJ concentrations (10%, 20%, 40% and 100%) on transepithelial electrical resistance and cell number was measured. The data suggested that a DJ concentration of 10% has no adverse effects on our cells. Therefore, this concentration was used for all subsequent experiments. The Caco-2 cell line was purchased from ATCC (LGC Standards GmbH, Wesel, Germany). The HT29-MTX cell line was given by Ms. N. Jablonowski from the research group, Microbial Pathogenicity Mechanisms (Leibniz Institute for Natural Product Research and Infection Biology, Hans Knöll Institute, Jena, Germany).

### 2.7. Barrier Integrity Analysis

The influence of digested and undigested CuO-NP on barrier integrity was assessed by measuring the transepithelial electrical resistance (TEER), measuring the permeability of FITC-dextran through the monolayers (Figure 1D) and visualizing the effects on the tight junctions with immunofluorescence staining of zonula occludens-1 (ZO-1).

#### 2.7.1. Transepithelial Electrical Resistance

The mono- and co-cultures were exposed to 1.3 × 10^−5^ M, 1.3 × 10^−4^ M and 1.3 × 10^−3^ M undigested and digested CuO-NP and CuCl_2_, cell culture medium (negative control; NC), 10% DJ (digestion control; DC) and 10 mM EGTA with 1% Triton-X-100 as a positive control (PC) for 24 h at 37 °C, 95% humidity and 5% CO_2_. The TEER values were measured using a chopstick electrode and an epithelial voltammeter (EVOM2; World Precision Instruments, Sarasota, FL, USA), in accordance with Ude et al. [2]. The electrodes were placed in the apical and basolateral compartments of the Transwell inserts, and the electrical resistance [Ω] was measured until the value remained constant. Relative TEER values were calculated by determining the TEER values before and after incubation.

#### 2.7.2. Permeability Assay

The mono- and co-cultures were exposed to 1.3 × 10^−5^ M, 1.3 × 10^−4^ M and 1.3 × 10^−3^ M digested and undigested CuO-NP and CuCl_2_, cell culture medium (medium control), 10% DJ (digestion control; DC) and 10 mM EGTA with 1% Triton-X-100 as PC for 24 h at 37 °C, 95% humidity and 5% CO_2_. To assess monolayer permeability, the FITC-dextran (10,000 MW) concentration in the basolateral compartment was measured after incubation for 24 h. For this, the cells were co-incubated with 1000 µg mL^−1^ FITC-dextran and the corresponding sample in phenol red-free DMEM for 24 h. Afterwards, 100 µL of basolateral medium was plated in triplicate in a black 96-well plate (Brand GmbH & CO. KG, Wertheim, Germany). The FITC fluorescence intensity was measured via spectral photometry (Synergy 2, BioTek, Berlin, Germany) at 528/20 nm. The FITC-dextran concentration was calculated using an FITC-dextran standard of known concentration. The permeability was assessed by comparing the calculated concentration to a blank value (FITC-dextran in a Transwell insert without cells) representing the highest possible FITC-dextran concentration after 24 h.

### 2.8. Cellular Uptake

To determine if the digestion process might have an effect on the rate of translocation of CuO-NP into Caco-2 cells, the uptake was assessed via electron microscopy. The Caco-2 cells were cultured in 25 cm^2^ cell culture flasks for 21 days. The differentiated Caco-2 cells were then exposed to cell culture medium and 1.3 × 10^−3^ M digested and undigested CuO-NP for 24 h. The cells were then washed twice with 5 mL PBS (Mg^2+^ and Ca^2+^) and fixed with 5 mL 2.5% glutaraldehyde solution in PBS for 1 h at room temperature in the cell culture flasks. Afterwards, the glutaraldehyde solution was removed, and the cells were washed twice for 20 min with 5 mL cacodylate buffer (100 mM at pH 7.2). After the first fixation and washing procedure, the cell-containing flasks were post-fixed with 1% (*w*/*v*) osmium tetroxide in cacodylate buffer for 2 h. After two washing steps with cacodylate buffer, the fixed cells were scraped off the culture dishes and pelleted 10 min at 500× *g*. The cell pellets were dehydrated in an ascending ethanol series and stained with 2% (*w*/*v*) uranyl acetate in 50% (*v*/*v)* ethanol. The samples were embedded in Araldite resin (Plano, Wetzlar, Germany) according to manufacturer’s instruction. Ultrathin sections of 70 nm thickness were cut using an ultra-microtome Ultracut S (Reichert-Jung, Wien, Austria) and mounted on Formvar-carbon coated 100 mesh grids (Quantifoil, Großlöbichau, Germany). The Ultrathin sections were stained with lead citrate for 10 min [58] and examined in a Zeiss CEM 902 A electron microscope (Carl Zeiss AG, Oberkochen, Germany). Images were obtained using a wide-angle dual speed 2 K CCD camera controlled by a Sharp:Eye base controller and operated by the Image SP software (camera, controller and software TRS, Moorenweis, Germany).

### 2.9. Analysis of Cell Viability

To compare the cytotoxic potential of undigested and digested CuO-NP, we assessed their impact on cell viability by propidium iodide (PI) staining and the induction of apoptosis by Annexin V-FITC staining using flow cytometry (Figure 1E). Prior to quantification of cell viability by flow cytometry, cell morphology was observed by light microscopy as well as fluorescence microscopy after the staining of cell nuclei and the cytoskeleton.

#### 2.9.1. Fluorescence Microscopy

To visualize potential effects of undigested and digested CuO-NP as well as CuCl_2_ on cell morphology, we performed cytoskeletal and nuclei staining of the cells directly after incubation. For this, cells were rinsed with PBS and fixed with 4% PFA for 20 min, followed by a permeabilization step with 0.1% Triton X-100 for 10 min. After a subsequent washing step, cells were incubated for 30 min in the dark in a humidifier with phalloidin (coupled with Alexa Fluor-488) to stain the cytoskeleton and DAPI to stain the cell nuclei. Afterwards, the cells were examined using fluorescence microscopy.

#### 2.9.2. Propidium Iodide Staining

The mono- and co-cultures were exposed to 1.3 × 10^−5^ M, 1.3 × 10^−4^ M and 1.3 × 10^−3^ M undigested and digested CuO-NP and CuCl_2_, cell culture medium and 10% DJ for 24 h at 37 °C, 95% humidity and 5% CO_2_. Afterwards, the cells were trypsinized for detachment from the Transwell insert, centrifuged (Heraeus Instruments, Hanau, Germany) at 600× *g*, washed with PBS and incubated with 5 µg mL^−1^ PI. The cytotoxic effects of digested and undigested CuO-NP were then determined by measuring the percentage of PI-stained cells by flow cytometry. Cell debris and cell duplets were excluded by a predetermined gating strategy.

#### 2.9.3. Annexin V-FITC Staining

The mono- and co-cultures were exposed to 1.3 × 10^−3^ M digested and undigested CuO-NP and CuCl_2_, cell culture medium, and 10% DJ for 24 h at 37 °C, 95% humidity, and 5% CO_2_. Subsequently, the cells were removed from the Transwell insert, centrifuged at 600× *g*, washed with 1 mL ice-cold PBS and centrifuged again at 600× *g*. The cell pellets were resuspended in 200 µL binding buffer. Afterwards, 5 µL Annexin V was added, and the cells were incubated for 15 min protected from light at 120 rpm. The fluorescence intensities of the FITC-labeled Annexin V were measured via flow cytometry. The fold changes of the median of the FITC fluorescence intensities were compared with those of the untreated control. The resulting data are expressed as normalized median fluorescence intensity (nMFI).

### 2.10. Statistical Analysis

All measurements were performed in triplicate for each sample with three independent biological replicates. To analyze particle stability, we used Student’s *t*-test for two independent samples with an assumed normal distribution. The statistical significance of differences between digested and undigested CuO-NP and the untreated medium control was analyzed by using one-way ANOVA followed by the Ryan–Einot–Gabriel–Welsch post hoc test (*) (SPSS Statistics 27 Premium, International Business Machines Corporation, Armonk, NY, USA). The statistical significance of differences between the cell culture models as well as between the digested and undigested CuO-NP was determined via Students *t*-test for two independent samples with an assumed normal distribution (#). Statistically significant differences are marked as #/* *p* < 0.05; ##/** *p* < 0.01; *** *p* < 0.001.

The results were plotted via Graphpad-Prism 6 (Graphpad Software Inc., San Diego, CA, USA) and Microsoft Office (Microsoft Corporation, Redmond, WA, USA). The graphic overview (Figure 1) was created in Inkscape.

## 3. Results

### 3.1. Physicochemical Characterization of CuO Dispersions

The influence of the simulated digestion process on the physicochemical properties of the investigated CuO-NP was determined using DLS, electrophoretic mobility and TEM. To account for particle transport in the employed in vitro Transwell system, which strongly depends on these properties [59], the delivered dose in comparison with the administered dose was calculated with the well-established distorted grid model [53].

#### 3.1.1. Size, Shape and Zeta-Potential of the Copper Oxide Nanoparticles

**Figure 2:**
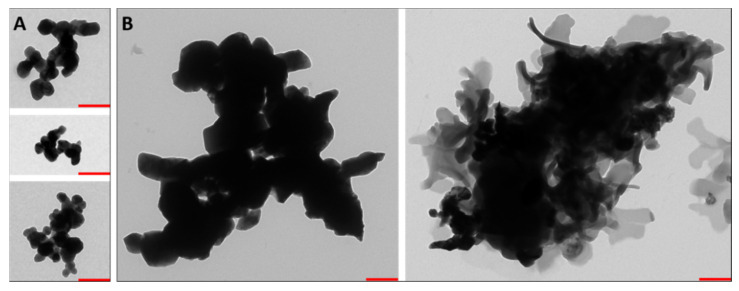
Impact of artificial digestion on the appearance of copper oxide nanoparticles (CuO-NP). Representative transmission electron microscopy images of agglomerates and/or aggregates of (**A**) undigested and (**B**) digested CuO-NP are shown. The red bars represent 250 nm.

The mean particle size of the CuO-NP (dissolved in cell culture medium) was analyzed by DLS and showed an increase in the mean particle or agglomerate size by about 50 nm after digestion (Table 1). The analysis of the TEM images also indicated a high degree of polydispersity of the undigested CuO-NP (Figure 2A) due to agglomeration/aggregation of the primary particles, which was also reflected in a relatively high polydispersity index of the DLS measurement (>0.2 for each sample, data not shown). In addition, qualitative particle analysis via TEM showed that, in addition to the average size, the shape of the primary particles had changed significantly in some cases (Figure 2B). The edges of the digested primary particles appear diffuse, compared with the undigested CuO-NP.

After digestion, CuO-NP showed a significantly lower (−6.3 ± 3.5 mV; *p* < 0.05) zeta potential compared with that of undigested CuO-NP (0.1 ± 0.5 mV; Table 1).

#### 3.1.2. Particle Transport Simulation

Interestingly, digestion of CuO-NP had no influence on the calculated delivered doses, which reflects the particles that reach the cells. About 75% (digested CuO-NP) and 79% (undigested CuO-NP) of initially added 100 µg mL^−1^ were delivered to the cell layer after 24 h of incubation (Table 1). Therefore, all effects detected by the following experiments were due to a comparable amount of particle material.

### 3.2. Intestinal Barrier Integrity

#### 3.2.1. TEER Value

**Figure 3:**
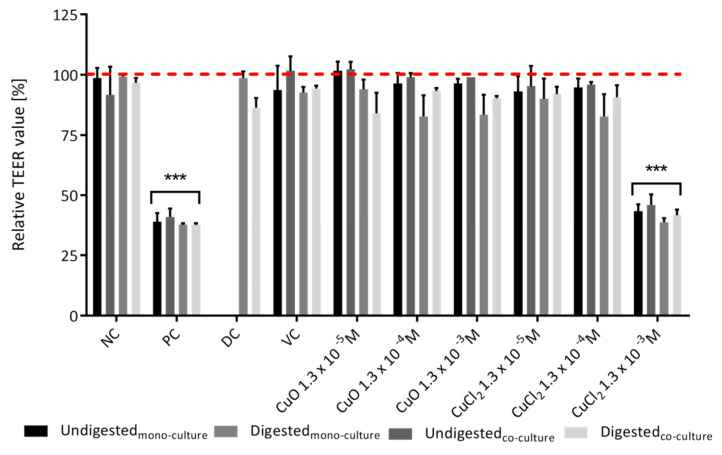
Impact of digested and undigested copper oxide nanoparticles on the barrier integrity of Caco-2 mono-cultures and Caco-2/HT29-MTX co-cultures. NC: negative control (cell culture medium); PC: positive control (10 mM EGTA and 0.1% Triton X-100); DC: digestion control (10% digestive juice in culturing medium); VC: vehicle control (0.05% BSA solution). The red line represents the initial TEER value before incubation. The statistical differences relative to the TEER values before treatment are marked as: *** *p* < 0.001. The error bars represent the standard deviation of three independent measurements.

To analyze the effect of CuO-NP on the barrier integrity of the cell layer, TEER values were measured before and after each treatment. No adverse effects of the particles were detectable, even after treatment with the highest concentration of 1.3 × 10^−3^ M (Figure 3). Neither digested nor undigested CuO-NP caused a significant decrease in TEER values, whereas a significant loss of relative TEER values of more than 50% for both mono- and co-cultures was observed for 1.3 × 10^−3^ M CuCl_2_ (Figure 3).

#### 3.2.2. Permeability

**Figure 4:**
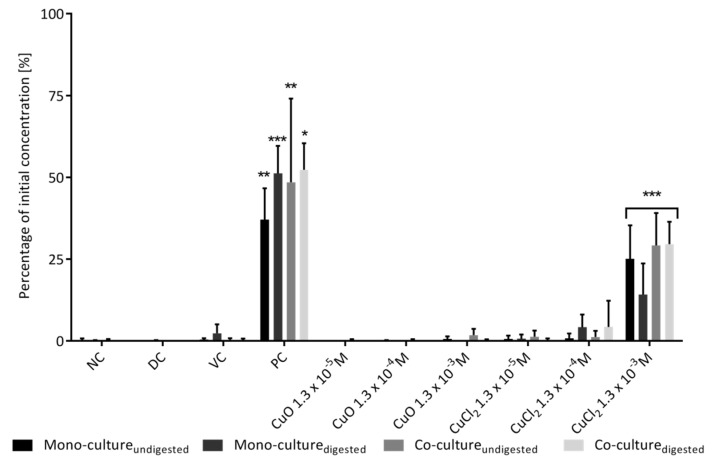
Permeability of the monolayers of Caco-2 mono-culture and Caco-2/HT29-MTX co-culture cells after incubation with digested and undigested copper oxide nanoparticles. The FITC-dextran concentrations after 24 h were normalized to the initial applied FITC-dextran concentration in the apical compartment, which was determined by incubating a Transwell insert without cells with FITC-dextran and measuring the basolateral concentration after 24 h. NC: negative control (cell culture medium); PC: positive control (10 mM EGTA and 0.1% Triton-X-100); DC: digestion control (10% digestive juice in culturing medium); VC: vehicle control (0.05% BSA solution). The statistical differences relative to NC are marked as: * *p* < 0.05, ** *p* < 0.01 and *** *p* < 0.001. The error bars represent the standard deviation of four independent measurements.

Cell layer permeability studies were performed by measuring the transport of FITC-dextran from the apical compartment to the basolateral compartment. Treatment with CuO-NP did not show any effect (Figure 4). Neither the digested nor the undigested CuO-NP caused a significant increase in the percentage of transported FITC-dextran in mono- and co-cultures. In contrast, incubation with 1.3 × 10^−3^ M CuCl_2_ resulted in up to 29% FITC-dextran of the initially applied FITC-dextran in the basolateral compartment (*p* < 0.001), which is in good agreement with the observed TEER data (Figure 3).

In summary, with the exception of ZO-1 staining (Supplementary Materials, Figure S3), the data collected show only minor disruption of barrier integrity after treatment with CuO-NP.

### 3.3. Determining Cellular Uptake by TEM

For the analysis of cellular uptake of the applied CuO-NP, sections of the cell layers were examined using TEM (data not shown). Almost no particle material could be found in the cells or on their surfaces. Isolated particles were only found in some cells whose structure was already disturbed.

### 3.4. Analysis of Cytotoxic Effects

#### 3.4.1. Integrity of the Cell Membrane

**Figure 5:**
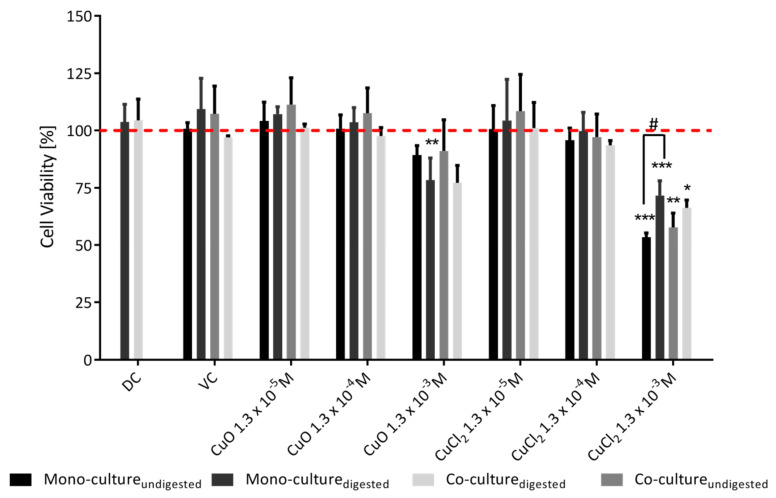
The effect of the digestion process on the cytotoxicity of copper oxide nanoparticles was determined via propidium iodide staining and flow cytometry. The cells of the Caco-2 mono-culture and the Caco-2/HT29-MTX co-culture were incubated with 10% digestive juice (DC), 0,05% BSA solution and CuO-NP/CuCl_2_ for 24 h. The mean vitality values were normalized to those of the negative control (NC), represented by the red line. The statistical differences between the samples and the NC are marked as * *p* < 0.05, ** *p* < 0.01 and *** *p* < 0.001, and the statistical difference between the digested/undigested samples is marked as # *p* < 0.05. The error bars represent the standard deviation of three independent measurements.

To determine the potentially harmful effects of CuO-NP on human intestinal cells, the impact on the integrity of the cell membrane as a marker of cell viability (PI assay) and the induction of apoptotic processes (Annexin V-FITC assay) was quantitatively analyzed using flow cytometry (Figure 5). No strong effects could be observed for CuO-NP with and without prior digestion. There was only a slight concentration-dependent decrease in cell viability for CuO-NP, while only incubation with 1.3 × 10^−3^ M digested CuO-NP resulted in a significant (*p* < 0.01) decrease in the viability of Caco-2 mono-cultured cells by approximately 22%. After incubation with 1.3 × 10^−3^ M digested and undigested CuCl_2_, the viability of mono- and co-cultures decreased by 29–47% (*p* < 0.05–*p* < 0.001). The undigested CuCl_2_ caused a significantly (*p* < 0.05) lower cell viability than the digested CuCl_2_ in the monoculture.

#### 3.4.2. Apoptosis Induction

**Figure 6:**
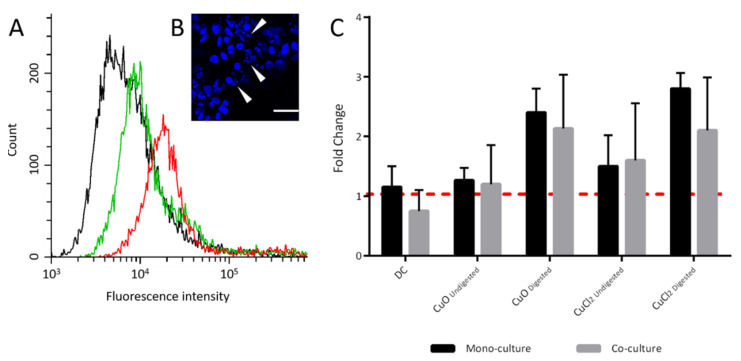
Detection of apoptosis induction by flow cytometry and fluorescence microscopy. (**A**) Histogram of Annexin V-FITC-stained Caco-2 mono-culture cells without (black) or with copper oxide nanoparticle (CuO-NP) treatment (green: undigested, red: digested). (**B**) DAPI-stained Caco-2 mono-culture cells after treatment with digested CuO-NP (white arrows indicate shrunken nuclei). (**C**) Fold change of apoptotic cells after treatment of Caco-2 mono- and Caco-2/HT29-MTX co-cultures with undigested and digested CuO-NP (1.3 × 10^−3^ M) or copper chloride (1.3 × 10^−3^ M). The samples were normalized to the untreated negative control (red line). All samples are means of three independent replicates and were measured in triplicate. DC: digestive control (10% digestive juice).

Immunofluorescence was used to investigate the effects of CuO-NP on the cell layers of the mono- and co-cultures (Supplementary Materials, Figures S4–S7). It was observed that there was an increased number of cells with shrunken nuclei (Figure 6B; Supplementary Materials, Figure S7). In order to determine whether these could be the result of apoptotic processes, the induction of apoptosis by CuO-NP was investigated. Annexin V-FITC staining, which is quantifiable by flow cytometry, was used as a relevant marker. The data (Figure 6A,C) show a clear trend of apoptosis induction after treatment with the digested samples, although the effects were not significant. Both the incubation with digested CuO-NP and with the ion control CuCl_2_ (1.3 × 10^−3^ M) caused an increase of up to 2.4-fold in the apoptosis rate, compared with that of the untreated control. The DC did not show any effects. It was also observed that digested and undigested CuCl_2_ caused apoptosis rates comparable to that of CuO-NP of equal concentration.

## 4. Discussion

In the present study, we assessed the impact of a static in vitro digestion on the physicochemical properties of CuO-NP and their effect on barrier integrity and cell viability of different models of the human intestine.

The impact of the in vitro digestion on the physicochemical properties of CuO-NP, depicted in Figure 2, could partially be due to the process of deglomeration in the gastric phase followed by re-agglomeration of the CuO-NP in the intestine, which was described for aluminum and silver nanoparticles in a previous study [50]. These changes in particle morphology could be explained by the pH-dependent modification of the protein corona surrounding the nanoparticles, which was described by Meziani and Sun [60]. Furthermore, crystalline structures appeared in the samples of the digested CuO-NP, indicating a possible reaction with the pH value-determining inorganic components (HCl or NaHCO_3_) as also previously described [50]. This interaction with the digestive juice could potentially influence the toxicity as well. Since the reaction of soluble Cu ions with these pH value determining components could attenuate toxic effects by reducing the Cu ion content of the media.

The surface charge of the digested CuO-NP (Table 1) can affect the bioavailability of the NP or their ability to come into contact with cells. Negatively charged particles were shown to interact less with the mucins, which were also negatively charged, on the epithelium and are thus more likely to come into contact with the cells, while positively charged particles could bind to mucins and thus be kept out of intestinal cells [61,62,63,64,65]. As a consequence, these influences could potentially affect the delivered dose of CuO-NP for in vitro experiments to varying degrees by modifying their physicochemical properties.

The digestion did not significantly impact the particle transport or size. Digested CuO-NP were slightly larger than undigested CuO-NP. Although larger particles might reach the intestinal cells faster, due to gravity, it has been shown that smaller particles usually tend to exhibit an elevated toxic potential, compared with larger particles of the same material, which is due, at least in part, to increased size-dependent cellular uptake of smaller NP [15,21,33,53,60,61,66,67,68].

Considering the impact of digested/undigested CuO-NP on the permeability, a comparison with the related scientific literature shows conflicting results. While Titma et al. [26] and Ude et al. [2] showed a 30–50% decrease in TEER values as a result of incubation with spherical CuO-NP, the work of Piret et al. [16] described a similar decrease only in the case of rod-shaped CuO-NP. The effects appear to be primarily dependent on the particle shape and not necessarily concentration-dependent, as Ude et al. [2] used almost equally high concentrations (12.68 μg cm^−2^) to those of Piret et al. [16], roughly corresponding to 11.16 μg cm^−2^. Therefore, it is probably reasonable to assume that the undigested polydisperse CuO-NPs used in our study were mainly spherical, as no significant effects occurred even at the highest concentration of 1.3 × 10^−3^ M (about 44.6 μg cm^−2^). This is underlined by the TEM images shown (Figure 2). Apart from the particle shape, the size of our CuO-NP (about 400 nm) could be a potential cause of the lower damage and permeabilization of the epithelium [15,26,33,61,68]. As shown in the literature, particles with larger mean sizes show reduced toxicity compared with smaller ones, causing significantly more adverse effects on cells in the case of oxidative stress, cell membrane disruption and alterations in the cytoskeleton, mitochondria and nucleus [15,33].

The slightly higher permeability of the co-culture, after incubation with 1.3 × 10^−3^ M CuCl_2_ (as seen in Figure 3 and Figure 4), did not represent a significant difference but is in line with the literature data. Lehner et al. [69] and Liu et al. [70] also observed an increase in permeability when comparing Caco-2 mono-cultures with Caco-2/HT29-MTX co-cultures with increasing percentages of HT29-MTX cells. This could be due to the fact that HT29-MTX cells form less tight junctions [71]. The TEM pictures suggest that potentially damaging effects on barrier integrity or cell viability are not necessarily caused by intracellular CuO-NP. Since the data on barrier integrity show that adverse effects were mainly induced by ionic copper, it can be concluded that most of the intracellular copper will probably be found in the form of copper ions. This is in good agreement with previously shown results for adverse effects of copper ions on Caco-2 cells [72,73]. A possible mechanism for this increased toxicity might be the generation of ROS by these free ions. The ROS generated by Cu ions can deplete intracellular GSH content and impair tyrosine kinase signaling, as summarized in Ferruzza et al. [73]. Since the percentage of solubilized Cu ions will probably be higher in CuCl_2,_ it makes sense to have an increased ion-mediated toxicity.

At the end of the digestion process, the digestive juices (with the nanoparticles) had a neutral pH value. As the release of Cu ions from CuO-NP is pH dependent, as shown by Studer et al. [74], this might also be a reason for a lower toxicity of CuO-NP compared to CuCl_2_.

The slightly negative charge of the digested CuO-NP, compared to undigested CuO-NP, did not seem to have any influence on the uptake process, even though an increase in uptake of up to three-fold has been described for negatively charged Cruciferin-NP in Caco-2/Caco-2-HT29-MTX cultures [75]. This result is in good agreement with the discussed correlation of particle size, agglomeration/aggregation behavior and cellular uptake. According to this, larger particles are taken up less frequently by the cells than smaller ones [8,46,76]. Nevertheless, it must be taken into account that the situation in vivo may vary considerably, as the composition and protein content of the incubation medium may differ greatly from that of the digestive juice in vivo [77,78]. The protein content in particular can have a considerable influence on cellular uptake due to the formation of a protein corona around the NP [14,77,79,80]. Since no particles were found in the ion control, it is probably safe to assume that there were no new particles formed in the course of the digestion.

The slightly increased toxicity of increasing concentrations of digested CuO-NP in comparison with that of the undigested CuO-NP on the Caco-2 mono-culture coincides with the concentration range used in a work by Henson et al. [51], even though a different cell model of the intestinal epithelium was used here. The similar cytotoxic potential of undigested and digested CuO-NP in this work could be due, among other things, to the fact that in vitro digestion does not significantly affect cellular uptake [47]. Nevertheless, the increased toxicity of digested CuO-NP could consequently be seen as a result of artificial digestion, since a small proportion of free copper ions could presumably be present after this process, compared with that in the undigested sample. This is because the investigated ion control (CuCl_2_) clearly showed that free ions were mainly responsible for the cytotoxic effect. In comparison with other studies on undigested CuO-NP on intestinal cells, again, some significant differences to our study can be seen. Several studies have shown that undigested CuO-NP had a strong cytotoxic effect on various epithelial cell models [20,21,24,26]. One possible reason for this difference could be the use of undifferentiated Caco-2 cells instead of differentiated cells in the aforementioned studies [20,26]. It has been shown that undifferentiated Caco-2 cells were more sensitive to various NPs [18,46,81], which in turn would result in higher toxicity levels. Studies on differentiated Caco-2 cells by Piret et al. [16] also showed little effect on viability, at least for spherical CuO-NP. Concentration-dependent effects after CuO-NP treatment were also described in other works, such as those by Henson et al. [51] and Li et al. [12], which are also recognizable in this work.

The enhanced apoptosis rate shown in Figure 6 could be the result of an increased expression in proapoptotic genes. Siddiqui et al. [38] and Alarifi et al. [82], for example, were already able to show this in other cell types after treatment with 10 µg mL^−1^ (1.3 × 10^−4^ M) CuO-NP. In these studies, the expression of the pro-apoptotic genes p53, bax and caspase 3 was upregulated, while anti-apoptotic bcl-2-expression was downregulated (for HepG2 cells). The trend toward an increased apoptosis rate with CuCl_2_ incubation is consistent with the data shown for greater reduction in barrier integrity and viability (Figure 3, Figure 4 and Figure 5). On the other hand, it was rather at odds with the current literature data, where dissolved ions had either lower or no significant detectable cytotoxic effect compared to CuO-NP [21,33,67,83,84]. Further studies on the induction of apoptosis by CuO-NP or dissolved Cu ions are therefore necessary to elucidate the concrete mechanism of toxicity.

## 5. Conclusions

In the present study, intensive attention was paid to the investigation of ingested CuO-NP and the influence of the digestion process on their toxicity in human intestinal cell models (mono- and co-culture). Only a slightly stronger effect of digested CuO-NP on the cells compared with that of undigested CuO-NP could be observed, whereas the presence of the additional mucus layer (Supplementary Materials, Figure S8) in the co-culture showed neither a positive nor a negative influence. In general, only weak effects could be detected for CuO-NP up to the maximum applied concentration of 1.3 × 10^−3^ M (100 µg mL^−1^), whereas the ionic form (CuCl_2_) had very clear effects on barrier integrity and cell viability at comparable concentrations. In summary, the digestion process, whose impact was analyzed by an artificial approach, had only a weak influence on the toxicity of CuO-NP in the studied concentration range (1.3 × 10^−5^–1.3 × 10^−3^ M or 1–100 µg mL^−1^) on a Caco-2 mono-culture or a Caco-2/HT29-MTX co-culture model. Further investigations need to analyze the fate of CuO-NP in biological fluids to determine the release of copper ions from the applied CuO-NP, since the ionic form seems to have a higher toxic relevance than the particulate form itself. Analytical methods, such as single-particle inductively coupled plasma mass spectrometry (SP-ICP-MS), could additionally help to further understand the mechanisms of particle behavior during digestion processes (e.g., dissolution behavior and agglomeration), which may lead to altered physicochemical particle properties and possibly altered toxicological effects.

## Figures and Tables

**Table 1:** Physicochemical properties of copper oxide nanopowder and copper oxide particle dispersions (before and after artificial digestion).

CuO-NP	SSA (m^2^ g^−1^)	dBET (nm)	ρENM (g cm^−3^)	dDLS (nm)	ζ-Potential (mV)	ADmax (µg mL^−1^)	DD (% of ADmax)
Undigested	3.4	272.2	6.48	396.3 ± 6.1	0.1 ± 0.5	100.0	79.0
Digested				450.2 ± 6.2	−6.3 ± 3.5	100.0	75.0

Data for the particle powder (SSA, dBET and ρENM) were provided by the manufacturer. Data for particle dispersions (dDLS, ζ-potential) are the means of three independent replicates, each of them measured in triplicate. SSA: specific surface area by nitrogen absorption (Brunauer–Emmett–Teller [BET] method); dBET: primary particle diameter determined from SSA; pENM: bulk material density; dDLS: mean hydrodynamic diameter; ζ-potential: zeta potential (surface charge); ADmax: maximum administered dose; DD: delivered dose.

## Data Availability

The datasets used and/or analyzed during the current study are available from the corresponding author on reasonable request.

## Supplementary Materials

The following supporting information can be downloaded at: https://www.mdpi.com/article/10.3390/toxics10030130/s1, Supplementary Table S1. Overview of the ingredients for in vitro digestion. Supplementary Table S2. Relevant input parameters for delivered dose calculation. Supplementary Figure S1. Influence of digestive juice on barrier integrity. Supplementary Figure S2. Influence of digestive juice on the cell count. Supplementary Figure S3. Immunofluorescent staining of tight junction protein ZO-1. Supplementary Figures S4–S6. Cytoskeleton staining with phalloidin. Supplementary Figure S7. Cell nuclei staining. Supplementary Figure S8. Mucus staining of mono- and co-cultures.
