# Supplementary material for: Artificial Digestion of Polydisperse Copper Oxide Nanoparticles: Investigation of Effects on the Human In Vitro Intestinal Co-Culture Model Caco-2/HT29-MTX
Source: Toxics. 2022 Mar 7;10(3):130. doi: 10.3390/toxics10030130 (PMC8955801; doi:10.3390/toxics10030130)
Supplement: Supplementary file 1 [file toxics-10-00130-s001.zip › toxics-1595969-supplementary.pdf]

toxics

MDPI

Supplementary Materials: Artificial Digestion of Polydisperse Copper Oxide Nanoparticles: Investigation of Effects on the Human In Vitro Intestinal Co-Culture Model Caco-2/HT29-MTX

Jevin Büttner, Thomas Schneider, Martin Westermann and Michael Glei

Composition of digestive juices

All components of the digestive juices employed are listed in Table S1 for each passage (mouth, stomach and small intestine).

Table S1. Composition of digestive juices for artificial digestion of copper oxide nanoparticles.

Digestive Juice	Chemicals/Components	Concentration
**Saliva**	NaCl	8.5 g l^{-1}
	α-Amylase	41.55 µl (1 U $µl^{-1}$)
	Mucin	3.0 g l^{-1}
**Gastric juice**	Pepsin	1.0 g l^{-1}
	KCl	0.7 g l^{-1}
	KH_2PO_4	0.27 g l^{-1}
	NaCl	2.9 g l^{-1}
	$CaCl_2*2H_2O$	0.5 g l^{-1}
**Small intestinal juice**	Bile	9.0 g l^{-1}
	Urea	0.3 g l^{-1}
	KCl	0.3 g l^{-1}
	$MgCl_2*6H_2O$	0.2 g l^{-1}
	$NaHCO_3$	1.0 g l^{-1}
	Pancreatin	9.0 g l^{-1}
	Trypsin	0.3 g l^{-1}

Additional remark: The volume for each sample was 15 ml saliva, 35 ml gastric juice and 50 ml small intestinal juice.

Simulation of particle transport *in vitro*

All relevant input parameters for the distorted grid model to simulate the transport behaviour of undigested CuO nanoparticles are listed in Table S2.

Table S2. Relevant input parameters for calculating the delivered doses of the copper oxide particle dispersion using the distorted grid transport model.

Input	Parameter	Unit	Value
Solvent	Viscosity	Pa s	0.725
	Density	g cm^{-3}	1.0037
	Temperature	°C	37.0
Particle	Concentration	mg cm^{-3}	0.1
Experiment	Incubation time	h	24
	Height of the media	mm	4.46
	Well area	mm^2	1.12
Simulation	Space	mm	0.005
	Time	s	1.0
Dissolution	Model	-	1
	Initial fraction	-	0.0
	Type dissolution rate	-	0
Adsorption	Stickiness	-	1
	Free particle fraction	-	0
	Dissociation constant	M	1.0 E-9

Additional remarks: The dissolution of copper ions was not taken into account, since stable particle dispersions over the incubation period of 24 h were generated as shown by DLS measurements. The input of diffusion and sedimentation coefficients was not used, since the calculation was done by using the corresponding particle size distribution for the tested material.

Exclusion of cytotoxic concentrations of digestive juice

The solutions employed in the static *in vitro* digestion approach are themselves cytotoxic due to the various ingredients (e.g. bile acids or inorganic salts). For experiments with digested nanoparticle dispersions, cytotoxic concentrations of this digestive juice were excluded. For this, preliminary experiments were performed, where decreasing concentrations of digestive juice were screened for their influence on transepithelial electrical resistance (TEER) values (barrier integrity) and their effects on the cell number remaining in our Transwell system, which was analysed by flow cytometry. The results are shown in Figure S1 and Figure S2. No adverse effects were observed for 10% digestive juice; therefore, we chose this dilution for all subsequent experiments.

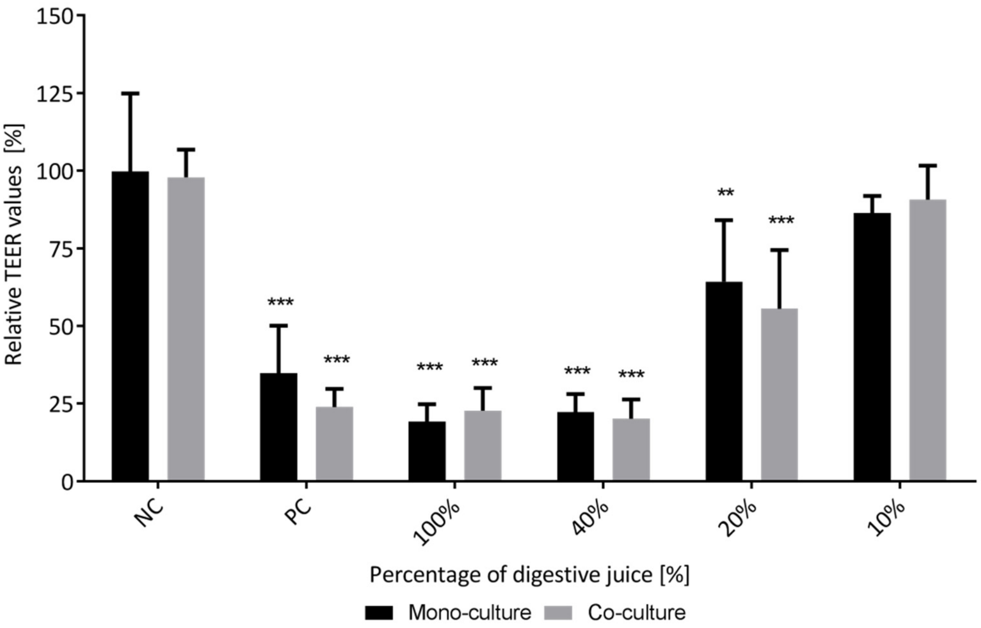

Figure S1. Influence of decreasing digestive juice concentrations on the barrier integrity of cell monolayers. Relative transepithelial electrical resistance (TEER) values are calculated by normalization of TEER values after incubation to TEER values before incubation. Results are shown for monoculture (Caco-2) and co-culture (80% Caco-2 and 20% HT29-MTX) cells. Data are means of triplicates of three independent experiments. NC = negative control (untreated); PC = positive control (10 mM EGTA). Significant values compared with NC are expressed as *** ($p < 0.001$) or ** ($p < 0.01$).

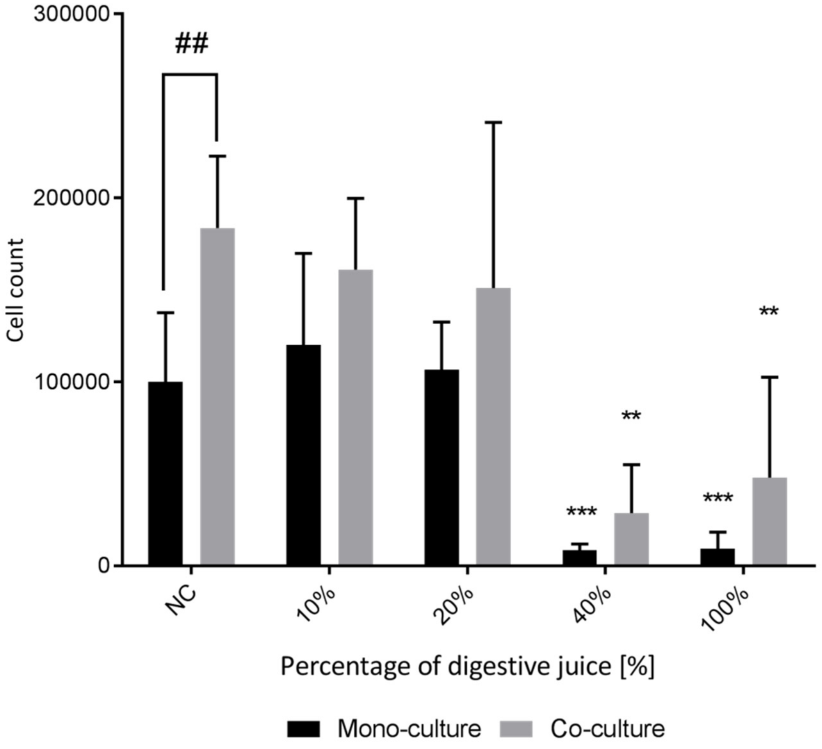

Figure S2. Influence of decreasing digestive juice concentrations on the cell count. Cells were analysed by flow cytometry in triplicate for three independent replicates. NC = negative control (untreated). Significant values compared with NC are expressed as *** ($p < 0.001$) or ** ($p < 0.01$). Significant differences between mono- and co-culture cells are marked with ## ($p < 0.01$).

Tight junction staining

In order to visualize possible adverse effects on the barrier integrity the impact of CuO-NP on tight junctions, using *in vitro* immunofluorescent staining of ZO-1, was investigated (Fig. S4).

The mono- and co-cultures were exposed to $1.3*10^{-5}$ M, $1.3*10^{-4}$ M and $1.3*10^{-3}$ M (respectively 1, 10 and 100 µg ml^{-1}) undigested and digested CuO-NP and $CuCl_2$, cell culture medium (medium control), DJ alone and 10 mM EGTA with 1% Triton-X-100 as positive control (PC) for 24 h at 37°C, 95% humidity and 5% CO_2. The cells were then washed twice with 200 µl PBS, fixed with 200 µl 4% paraformaldehyde for 20 minutes and washed again with 200 µl PBS. To permeabilize the cell membranes, the mono- and co-cultures were incubated with 200 µl 0.1% Triton-X-100 for 10 minutes and washed twice with 200 µl BSA solution (1% BSA in PBS). To block unspecific binding sites, the cells were then incubated with 200 µl BSA solution for 1 h on a shaker (neoLab Migge GmbH, Germany). After removal of the BSA solution, 200 µl of the primary antibody (anti-zona occludens 1 (ZO1) tight junction protein antibody; rabbit) was added to each well. The cells were incubated overnight at 4 °C on a shaker. The next day, the antibody solution was removed, the cells were washed twice with 200 µl PBS and incubated with 200 µl of the secondary antibody (goat anti-rabbit IgG conjugated to Alexa Fluor 568) at room temperature in the dark for another hour. After this, the cells were washed twice with 200 µl PBS and incubated with 0.1 µg ml^{-1} DAPI for 1 h with shaking. Afterwards, the Transwell inserts were placed under a confocal laser scanning microscope (Carl Zeiss AG, Germany) to visualize the tight junction protein ZO-1 and the cell nuclei.

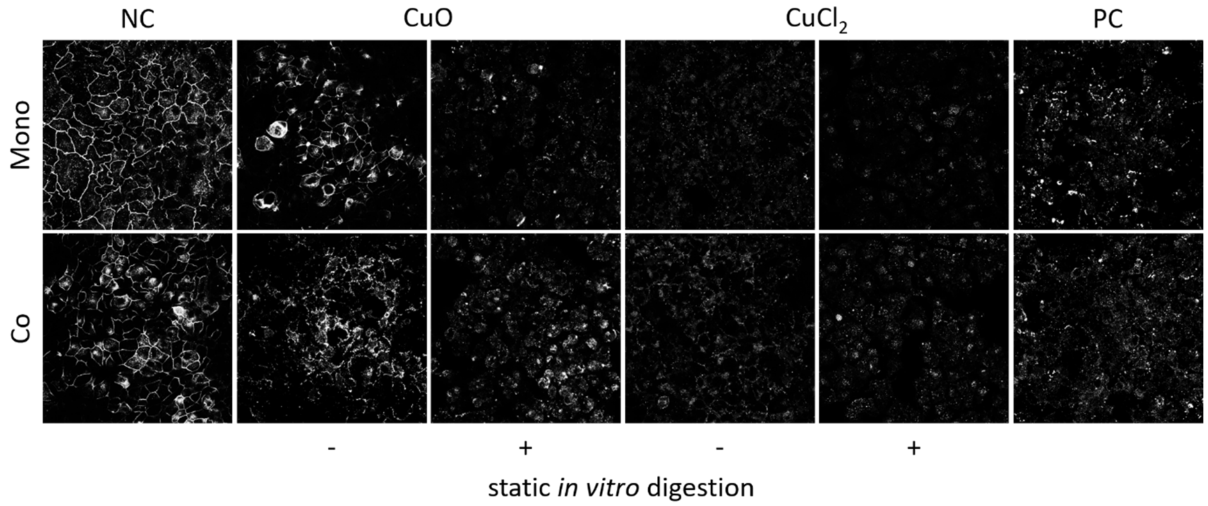

Figure S3. Immunofluorescent staining of tight junction protein ZO-1 (white) after treatment of Caco-2 mono- (Mono) and Caco-2/HT29-MTX co-cultures (Co) with undigested (-) or digested (+) copper oxide (CuO) nanoparticles ($1.3*10^{-3}$ M) and copper chloride ($CuCl_2$, $1.3*10^{-3}$ M). NC: untreated control. PC: 10 mM EGTA.

For untreated cells, a continuous connection between all cells clearly showed intact tight junctions, indicating fully functional cell layer integrity [3]. For undigested CuO-NP, this cellular connection was only partly interrupted for both mono- and co-cultured cells. In contrast, for digested CuO-NP as well as $CuCl_2$ (undigested and digested), no intact cellular connections could be observed, indicating strong degrading effects on the ZO-1 protein after treatment.

Microstructure Analysis

Cytoskeleton staining with phalloidin was performed to visualize cell-cell contacts of the mono-/co-culture after incubation with CuO-NP/$CuCl_2$.

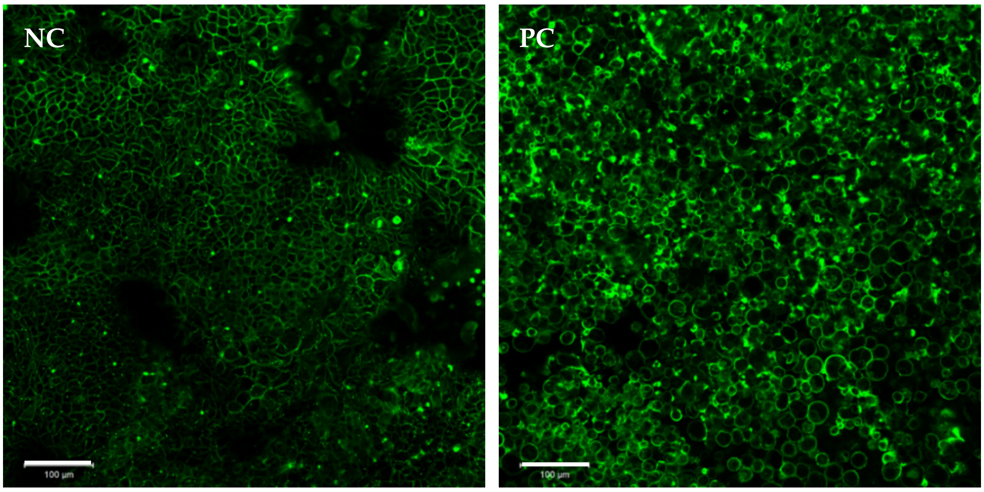

Figure S4. Cytoskeleton staining with phalloidin. Depicted are representative images of differentiated cells from the Caco-2/HT29-MTX co-culture after incubation with the negative control (culturing medium) = NC and after incubation with the positive control (EGTA and Triton-X-100) = PC. The white bar represents 100 μm.

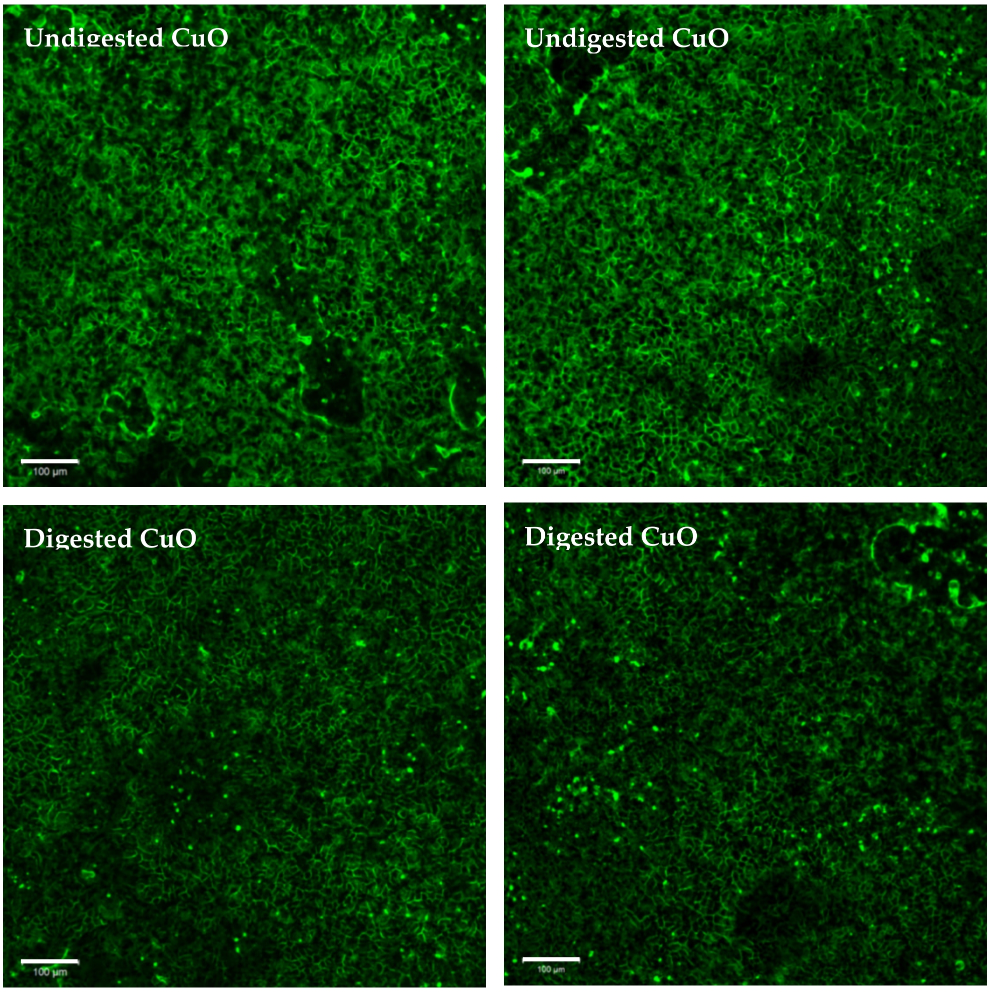

Figure S5. Cytoskeleton staining with phalloidin. Depicted are representative images of differentiated cells from the Caco-2 mono-culture (left) and the Caco-2/HT29-MTX co-culture (right) after incubation with undigested/digested CuO-NP ($1.3*10^{-3}$ M). The white bar represents 100 µm.

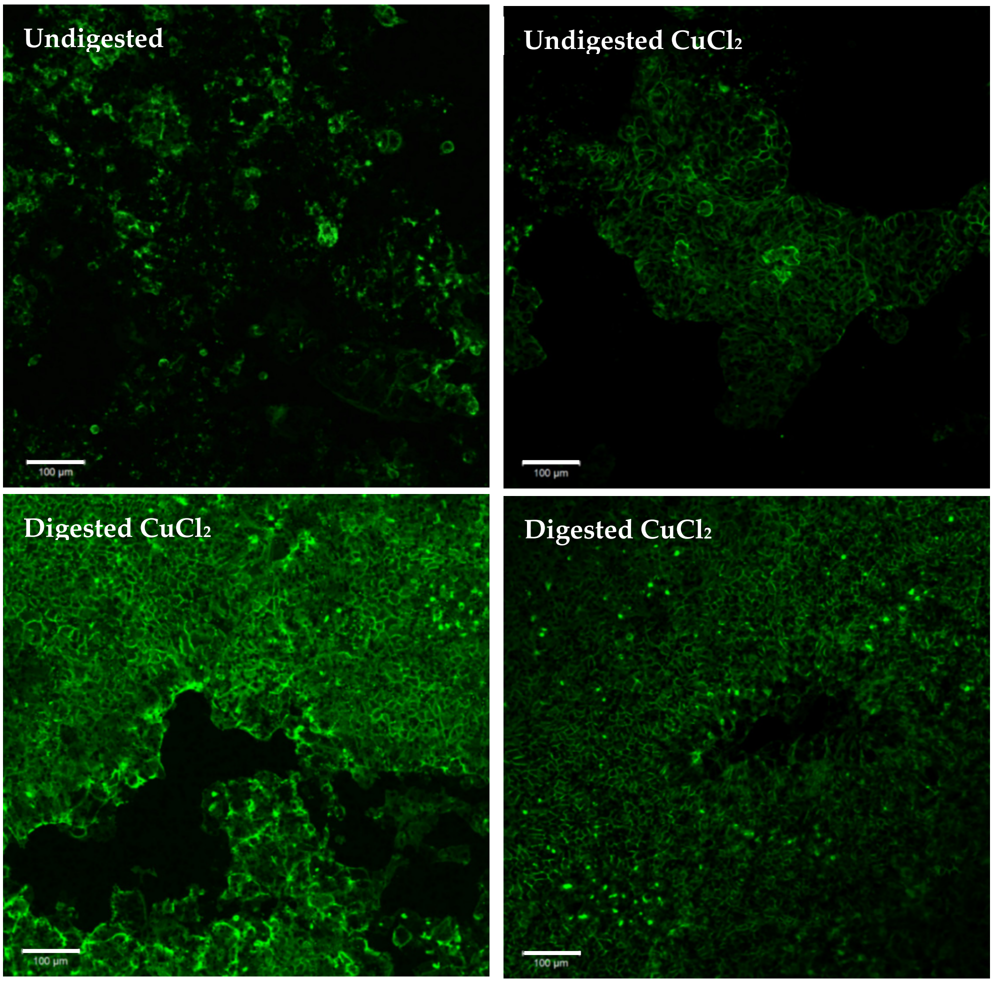

Figure S6. Cytoskeleton staining with phalloidin. Depicted are representative images of differentiated cells from the Caco-2 mono-culture (left) and the Caco-2/HT29-MTX co-culture (right) after incubation with undigested/digested $CuCl_2$ ($1.3*10^{-3}$ M). The white bar represents 100 µm.

Cell nuclei staining.

To visualize an exemplary impact on the microstructure of cell nuclei a DAPI cell staining of the co-culture after incubation with undigested/digested $CuCl_2$ and of untreated cells was performed. Since 1.3*10^{-3} M of $CuCl_2$ showed the strongest effect on cell viability, we chose these cells to visualize potential effects on the nuclei.

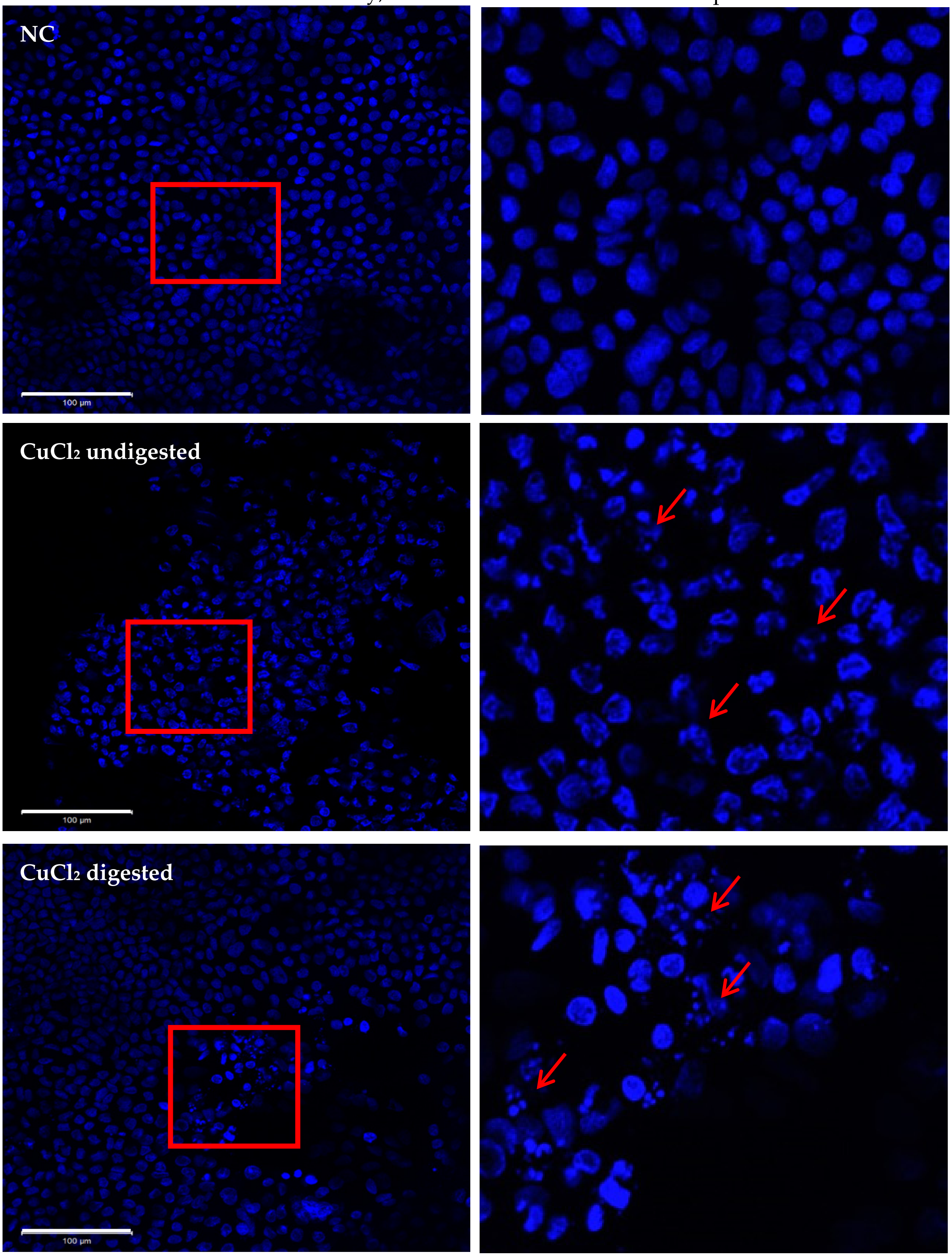

Figure S7. Cell nuclei staining. Depicted are cells without treatment (negative control (NC) = culturing medium) and cells after incubation with 1.3*10^{-3} M of undigested/digested $CuCl_2$. The images in the right column are magnifications of the corresponding red rectangles. The red arrows indicate micronuclei/fragmented nuclei.

Mucus staining

In our work, we compared the toxicity of undigested and digested copper oxide nanoparticles on Caco-2 mono-cultures and Caco-2/HT29-MTX co-cultures. Since the presence or absence of mucus might be important for potential treatment effects, we proved the presence of mucus. This was achieved by performing a Alcian Blue mucus staining protocol in accordance to [2] and [4]. Caco-2 cells and HT29-MTX cells as a mono-culture and as a co-culture (80:20) were seeded into a 6-well plate to grow and differentiate for 21 days [1]**.** Afterwards, the cell medium was removed, and the cells were washed with phosphate buffered saline (PBS) solution. The cells were then fixed by adding 1 ml methacarn solution (60% methanol, 30% chloroform and 10% acetic acid) at 4 °C for 1 h. The cells were then rinsed with ddH$_2$O and incubated with 1 ml 3% acetic acid for 3 minutes. Afterwards, 1 ml of 1% Alcian blue solution was added for 30 minutes. The excess Alcian blue was removed by rinsing with ddH$_2$O before the extent of mucus staining was analysed via bright-field microscopy (Fig. S3 of additional file 1). As shown in Figure S3, stained mucus (blue) was detectable in our co-culture as well as in our positive control (mucus-producing HT29-MTX only), while no mucus was detected for Caco-2 mono-cultured cells.

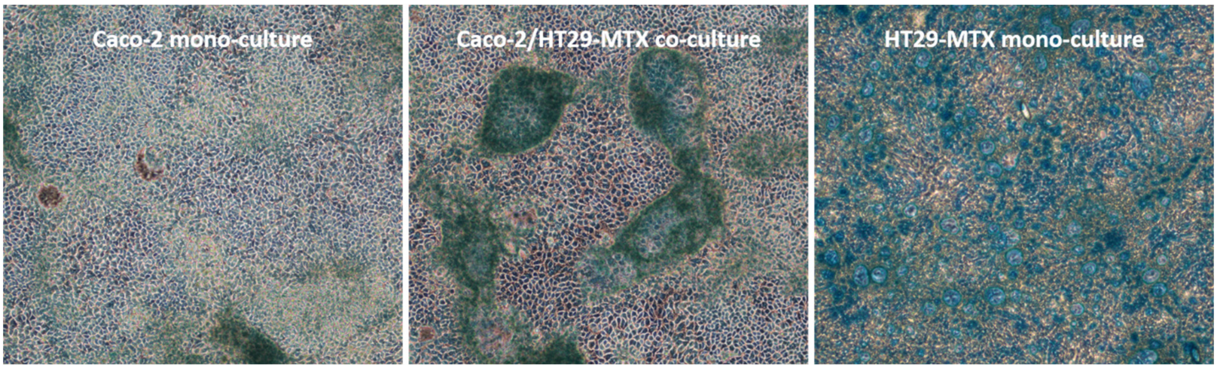

Figure S8. Mucus detection by Alcian blue staining of mono- and co-cultures. The HT29-MTX mono-culture was used as a positive control to compare the level of mucus production in the Caco-2 mono-culture and the co-culture (80% Caco-2 and 20% HT29-MTX).

References

1. Stock, V.; Böhmert, L.; Lisicki, E.; Block, R.; Cara-Carmona, J.; Pack, L.K.; Selb, R.; Lichtenstein, D.; Voss, L.; Henderson, C.J. Uptake and effects of orally ingested polystyrene microplastic particles in vitro and in vivo. *Archives of Toxicology* **2019**, *93*, 1817–1833.
2. Lehner, R.; Wohlleben, W.; Septiadi, D.; Landsiedel, R.; Petri-Fink, A.; Rothen-Rutishauser, B. A novel 3D intestine barrier model to study the immune response upon exposure to microplastics. *Archives of Toxicology* **2020**, 1–17.
3. Fanning, A.S., Jameson, B.J., Jesaitis, l.a., Anderson, J.M. The tight junction protein ZO-1 establishes a link between the transmembrane protein occludin and the actin cytoskeleton*. J Biol Chem*, **1998**. *273(45),* 29745–29753.
4. Béduneau A., Tempesta C., Fimbel S., Pellequer Y., Jannin V., Frédéric D. Lamprecht A. A tunable Caco-2/HT29-MTX co-culture model mimicking variable permeabilities of the human intestine obtained by an original seeding procedure. *Eur J Pharm Biopharm*, **2014**. *87(2),* 290–298.
